# Cell-to-cell transmission of HIV-1 between CD4^+^T-cells involves clathrin-mediated endocytosis

**DOI:** 10.1186/1742-4690-8-S2-P63

**Published:** 2011-10-03

**Authors:** Richard D Sloan, Björn D Kuhl, Jan Münch, D Aaron Donahue, Mark A Wainberg

**Affiliations:** 1 McGill University AIDS Centre, Lady Davis Institute, Montreal, Quebec, H3T 1E2 Canada; 2 Institute of Molecular Virology, Ulm University Medical Center, Ulm 89081, Germany

## Background

HIV-1 infection of CD4^+^T-cells occurs through cell free infection or through cell-cell contacts. Cell-to-cell transmission is the predominant mode of viral spread in lymphoid tissue and cell culture. Cell free infection of HIV-1 has recently been described to involve clathrin- and dynamin-mediated endosomal uptake of virus. There are also accounts of cell-to-cell transfer of virus involving endosomal uptake, but the precise details of this process remain unclear. Here, we asked what forms of endocytosis might be involved in such cell-to-cell transmission.

## Materials and methods

Cells were infected with GFP reporter virus bearing either CXCR4 or CCR5 tropic or the VSV-G envelope. Cell tracker stained target cells were mixed with infected donor cells. Target cells were pretreated with drugs or antibodies to inhibit HIV-1 entry or replication, or were treated to inhibit various forms of endocytosis. Single target cells were then assayed by flow cytometry for the presence of intracellular viral p24 shortly after co-culture, or for expressed GFP at a later time point to allow the distinction between transfer of virus (p24) and transmission of virus (expression of viral GFP).

## Results

We found that inhibiting CD4 binding prevented transfer of virus to the target cell and hence transmission of viral genes. In contrast, inhibiting the viral entry cascade downstream of CD4 binding, i.e. coreceptor binding or membrane fusion, had little capacity to prevent transfer of viral p24 to the target cell despite such treatment ultimately preventing the expression of viral genes. The same pattern of results was seen with both R5 and X4 tropic virus. Secondly, inhibition of clathrin-mediated endocytosis and dynamin-mediated endosome scission both prevented transfer and transmission of virus, whilst inhibition of macropinocytosis and caveolae-mediated endocytosis inhibited neither transfer nor transmission of virus.

## Conclusions

Collectively, our results show an involvement of clathrin-and dynamin-mediated endocytosis in cell-to-cell transmission of HIV-1 between T-cells and also suggest that events of the viral entry cascade downstream of CD4 binding can occur from within endosomes.

